# Morphological Changes in Lamellar Macular Holes According to SD-OCT Examination over a Long Observation Period

**DOI:** 10.3390/diagnostics11071145

**Published:** 2021-06-23

**Authors:** Magdalena Kal, Izabela Chojnowska-Ćwiąkała, Mateusz Winiarczyk, Monika Jasielska, Jerzy Mackiewicz

**Affiliations:** 1 Collegium Medicum of Jan Kochanowski University in Kielce, Ophthalmic Clinic of the Voivodeship Hospital in Kielce, 25-369 Kielce, Poland; kalmagda@gmail.com; 2 Institute of Medical Science of Jan Kochanowski University in Kielce, 25-369 Kielce, Poland; i.cwiakala@gmail.com; 3 Department of Vitreoretinal Surgery, Medical University of Lublin, 20-079 Lublin, Poland; mhjasielska@gmail.com

**Keywords:** lamellar macular hole, LMH, OCT, Max RT temporal, Max RT nasal

## Abstract

Background: The aim of this study was to evaluate the quantitative morphological changes in lamellar macular holes (LMHs) based on SD-OCT examinations and to assess the correlations among minimal retinal thickness (MRT), reading vision (RV), and best corrected visual acuity (BCVA) over a 36-month follow-up period. Methods: A group of 40 patients (44 eyes) with LMH was evaluated, with an average age of 69.87 (SD = 10.14). The quantitative parameters monitored in the follow-up period (at 0, 3, 6, 12, 18, 24, 30, and 36 months) were tested for normality of distribution by Shapiro–Wilk and Kolmogorov–Smirnov tests. Results: The RV and BCVA values were stable, and no significant changes were found at any of the check-ups during the 36-month follow-up period (BCVA *p* = 0.435 and RV *p* = 0.0999). The analysis of individual quantitative LMH parameters during the 36-month follow-up period did not demonstrate statistically significant differences: MRT (*p* = 0.461), Max RT temporal (*p* = 0.051), Max RT nasal (*p* = 0.364), inner diameter (ID) (*p* = 0.089), and outer diameter (OD) (*p* = 0.985). Conclusions: The observations at 0, 6, 12, 18, 24, 30, and 36 months revealed moderate and significant correlations between RV and MRT. No significant correlation between BCVA and MRT was observed.

## 1. Introduction

**Figure 1:**
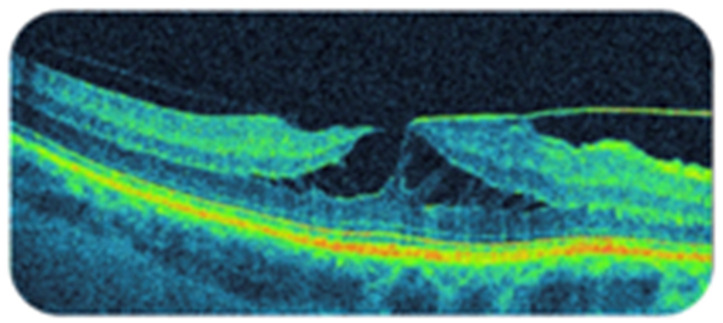
A lamellar macular hole. The presented image is an LMH with a major tractional component, with a “moustache” appearance, according to Govetto et al.

The first case of a non-full-thickness macular hole was described by Donald Gass, in 1975, [1] and was related to Irvine–Gass syndrome [1,2]. With the development of swept source OCT (SS-OCT), it became the gold standard for LMH diagnostics. Traditionally, the presence of an LMH according to an OCT examination has been described as a partial-thickness macular hole with an epiretinal membrane (ERM) and irregular contour of the fovea, located below the outer plexiform layer and the separation of the inner and outer layers within the central retina [3,4,5] (Figure 1). All forms of LMHs were treated as variations of one disease until 2016, when Govetto et al. divided LMHs into tractional and degenerative LMHs, suggesting that they were two different clinical entities [6]. Additionally, the presence of a typical ERM was questioned, with the suggestion that it should be named lamellar hole-associated epiretinal proliferation (LHEP) in cases with membrane contiguous with mid-retinal layers where the contractile component was absent [7,8,9]. All these changes in the understanding of LMHs have affected the current treatment of what was previously perceived as a homogenous disease.

Posterior vitreous detachment (PVD) probably plays the most significant role in the development of idiopathic non-full-thickness macular holes (NFTMHs). During its development, fragments of the internal limiting membrane (ILM) are detached, which stimulate the proliferation of Müller cells and the creation of the epiretinal membrane [10]. By shrinking, the membrane creates anterior–posterior vitreomacular traction, tangential to the surface of the retina. LMH may be idiopathic or develop after cataract surgery or with nearsightedness, uveitis, AMD, or retinal detachment [11,12,13,14,15].

The treatment of LMHs remains controversial. While the conservative approach has been to simply monitor LMHs, as they tend to be stable over the long term, there are studies that have suggested certain benefits from surgical intervention [16]. Especially in a time of rapid developments in the field of vitrectomy instruments and smaller gauges leading to lower rates of complications and faster visual recovery, surgical approaches seem to have become preferable for LMH treatment [17]. Vitrectomy with internal limiting membrane (ILM) peeling appears to be more effective for tractional LMHs than for degenerative LMHs, which are associated with a higher incidence of postoperative full-thickness macular holes [16,18,19,20,21]. In addition, LMHs with better preoperative RPE integrity seem to have better surgical outcomes [22].

In our study, we aimed to find correlations between the morphological changes in LMHs and BCVA and RV. To our knowledge, to date, there is limited evidence of an association of RV with MRT in LMHs. In our study, we aimed to find correlations between the morphological changes in LMHs and BCVA and RV. When planning a surgical intervention, especially RV and the presence of metamorphopsia should be assessed, as they are most likely to change after vitrectomy.

## 2. Materials and Methods

This retrospective, observational study of consecutive patients diagnosed with lamellar macular holes and seen by one retinal specialist (J.M.) was conducted at the Vitreoretinal Surgery Department, Medical University of Lublin, Poland. Since an OCT examination is part of the routine clinical procedure, no approval from an ethical committee was needed. Informed consent was obtained from each patient. The study was conducted according to the Declaration of Helsinki.

The exclusion criteria for the study group included the following diseases: diabetic retinopathy, central retinal vein occlusion, past eye injuries, past retinal surgery, high myopia, past intraocular inflammation, age-related macular degeneration, and glaucoma. In addition, patients who had an insufficient number of check-ups were excluded.

For each qualified patient, a complete ophthalmological examination was conducted, which included the following:

- A thorough examination of ophthalmological history and family history of general illnesses;
- An RV and BCVA examination; RV was checked in a sitting position with a chart at a distance of 33 cm, BCVA was checked on a logMAR ETDRS 5 × 5 chart (Sloan letters, CDHKNORSVZ) at a standard distance of 4 m and chart luminance set at 160 cd/m^−2^, and visual acuity was tested by subjective refraction;
- An evaluation of the anterior segment of the eyeball;
- An evaluation of the posterior segment of the eyeball via a slit lamp with the use of a 78 D Volk lens;
- The measurement of intraocular pressure by “air-puff” tonometry (Reichert, Depew, NY 14043, USA);
- An evaluation of the posterior segment of the eyeball via SD-OCT (Copernicus HR, Optopol, Poland).

**Figure 2:**
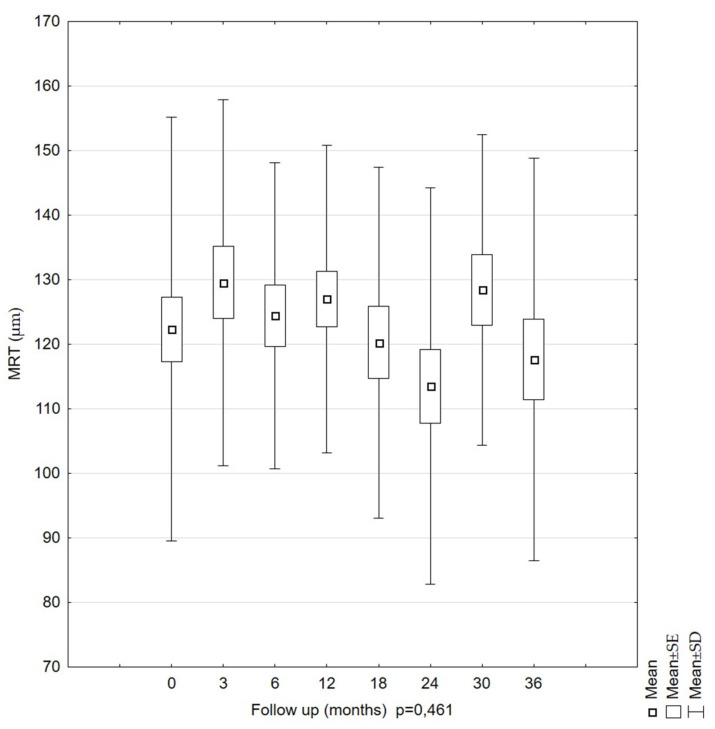
Mean minimal retinal thickness (MRT) values in LMH patients during the 36-month follow-up period. No significant changes were observed, showing that LMHs were stable over this period.

The morphological changes in a retina with LMH were assessed with an SD-OCT device (Copernicus HR, Optopol, Poland), which was used to perform spectral tomography of the central retina with a light wavelength of 840 nm and spectrum width of 50 nm. The axial (longitudinal) resolution of the device was 6 µm, the transverse resolution was 12–18 µm, and the width of the tomogram window was 2 mm. The measurement rate was 25,000 A-scans per second, with a maximum scanning width of 10 mm. The maximum number of A-scans per B-scan was 10,500. The dimensions of the obtained image were 570 × 650 × 670 µm (width × height × length), and the maximum size of the scanning area was 12 × 9 mm. The higher infrared permeability enabled the examination of cataract patients. The OCT examinations were performed at 0, 3, 6, 12, 18, 24, 30, and 36 months of the follow-up period. The following parameters were evaluated with the use of the SD-OCT Copernicus HR device (Figure 2):

- The manual measurement of the MRT (minimal retinal thickness under the fovea);
- The manual measurement of the Max RT (maximum retinal thickness) at a distance of 750 µm from the foveola in the temporal (Max RT temporal) and nasal (Max RT nasal) parts of the macula;
- The ID (inner diameter) manual measurement of the distance between the edges of the macular hole within the inner retinal layer;
- The OD (outer diameter) manual measurement of the outer diameter of the lamellar macular hole within the outer retinal layers.

### Statistical Analysis

With regard to quantitative features assessed during the follow-up period of the study (0, 3, 6, 12, 18, 24, 30, and 36 months), the normality of the distribution was verified by the Shapiro–Wilk test. The statistical results were matched to the distribution of traits.

The characteristics of the analyzed quantitative traits, $\bar{x}$(SD) and Me(IQR), were calculated for each examination. The Friedman test was used to assess the variation of parameters over time for traits significantly deviating from the normal distribution. For the traits whose distributions were normal, one-way ANOVA was used for systems with repeated measurements.

The standard deviation (SD) was used to measure the dispersion around the mean value, and the interquartile range (Q1–Q3), for the median.

Spearman’s rank correlations were used to determine the relationships among RV, BCVA, and MRT during the 36-month follow-up period for the LMH patients. Statistical significance was considered at *p* ≤ 0.05.

## 3. Results

The study group comprised 40 patients (44 eyes) with LMHs, of whom 35 were women (87.50%) and five were men (12.50%). The average age was 69.87 (SD = 10.14). Myopia was found in 19 eyes (43.18%), and hyperopia, in 14 eyes (31.82%), whereas emmetropia was found in 11 eyes (25.0%). The refraction of the patients ranged from −5.0 to +5.0 D. The number of phakic patients was 36 (81.81%), while eight patients were pseudophakic (18.19%). The follow-up time was 36 months, with examinations performed at 0, 3, 6, 12, 18, 24, 30, and 36 months.

**Figure 3:**
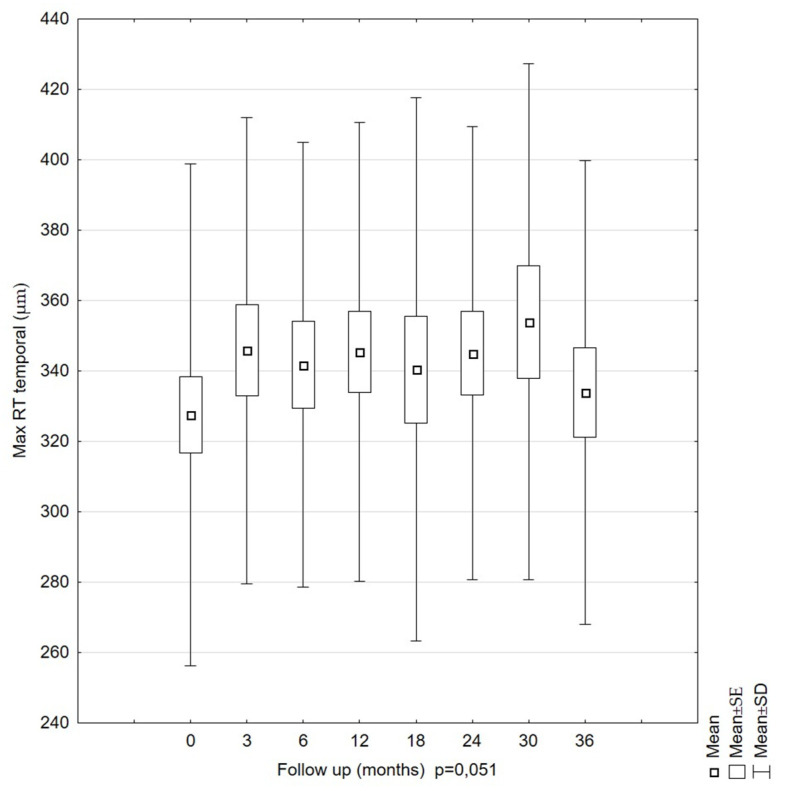
Average values of maximal retinal thickness (Max RT) for the temporal area of patients during the 36-month follow-up period. The Max RT temporal value showed no statistically significant changes over this period.

After the SD-OCT examination, the quantitative parameters of the LMHs were analyzed. The analysis of the MRT values in the study group during the 36-month follow-up period showed no statistically significant differences (*p* = 0.461) (Figure 3). The mean values of the MRT measurement were 122.35 µm (SD = 32.85) at the first examination and 117.68 µm (SD = 31.18) at the last examination. This parameter was stable over time (*p* = 0.461). The mean values were similar in each examination, as were the errors of the mean. The 95% confidence intervals for the mean values were the highest for the second measurements (at 3 months).

**Figure 4:**
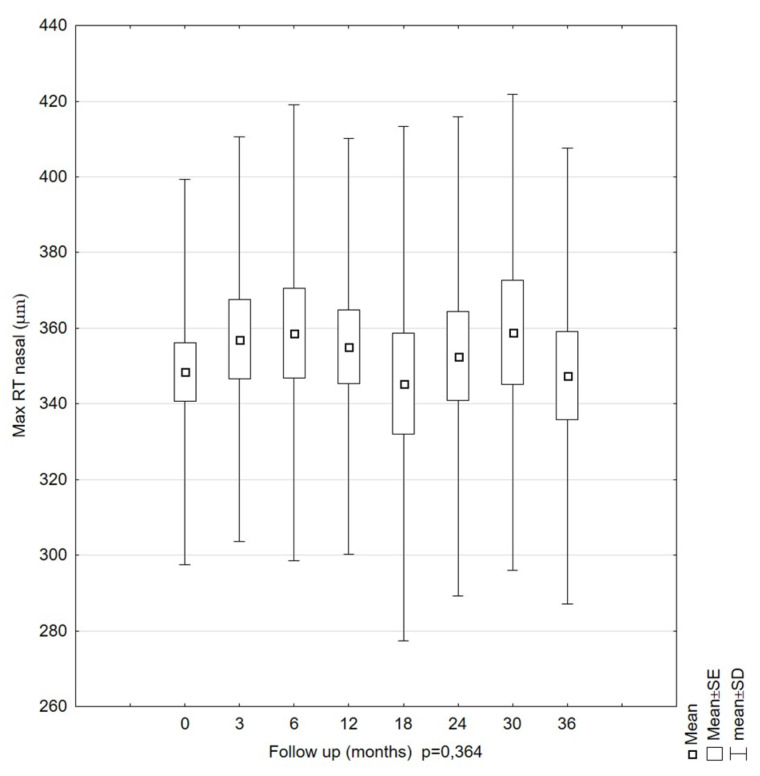
Mean values of the maximal retinal thickness (Max RT) for the nasal area parameter of patients during the 36-month observation period. The Max RT nasal value showed no statistically significant changes over this period.

The mean Max RT temporal measurements were 327.58 µm (SD = 71.29) at the first examination and 333.39 µm (SD = 65.96) at the last examination. This parameter did not change significantly over time (*p* = 0.051). The highest Max RT temporal value was found at 30 months, and the lowest values, at 0 and 36 months. The standard errors for subsequent measurements were similar (Figure 4).

**Figure 5:**
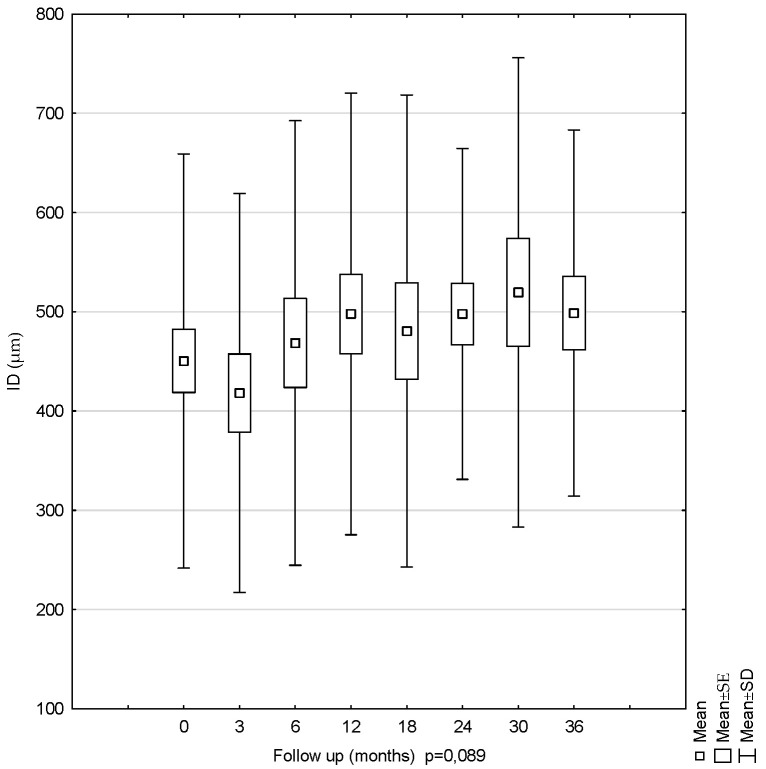
Mean internal diameter (ID) values for the 36-month follow-up period. The ID showed no statistically significant changes over this period.

The mean Max RT nasal measurements were 348.47 µm (SD = 50.90) at the first examination and 347.41 µm (SD = 60.28) at the last examination. No statistically significant changes were found (*p* = 0.364). The highest values for this parameter were recorded at 6 and 30 months, and the lowest, for the first measurement (at 0 months). The standard errors of the means were similar, and the confidence intervals indicated similar ranges of variability for the individual patient observations (Figure 5).

**Figure 6:**
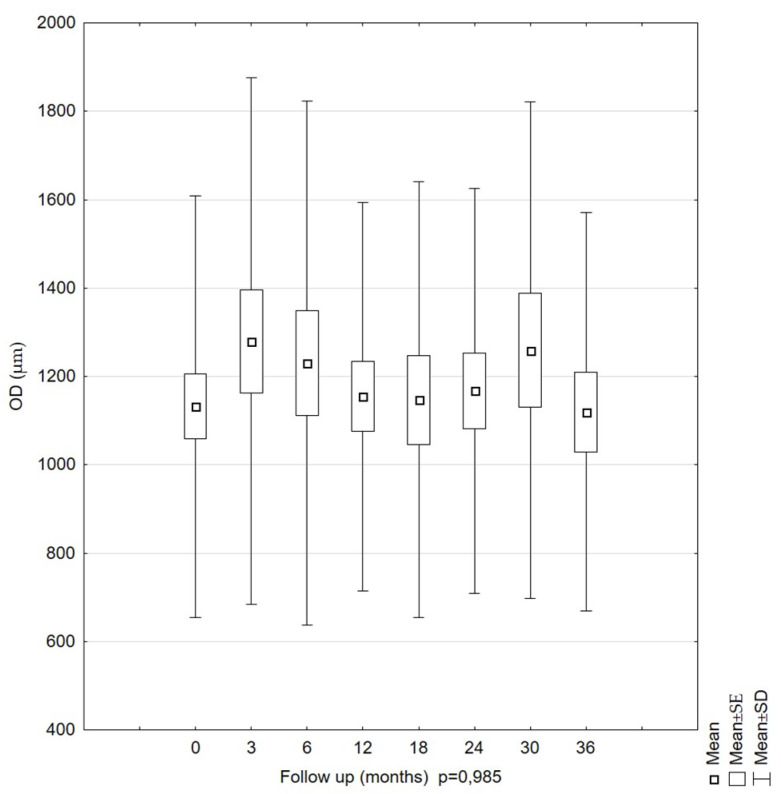
Mean values of the outer diameter (OD) parameters in LMH patients during the 36-month follow-up period. The OD showed no statistically significant changes over this period.

The mean ID measurements were 450.42 µm (SD = 208.60) at the first examination and 498.76 µm (SD = 184.52) at the last examination. Between 0 and 36 months of the follow-up, a slight increase in the ID value was observed. The lowest mean value was observed at 3 months, and the highest, at 30 months. However, the observed changes were found to be statistically insignificant (*p* = 0.089). The error values for the mean values during the 36-month follow-up period remained similar (Figure 6).

**Figure 7:**
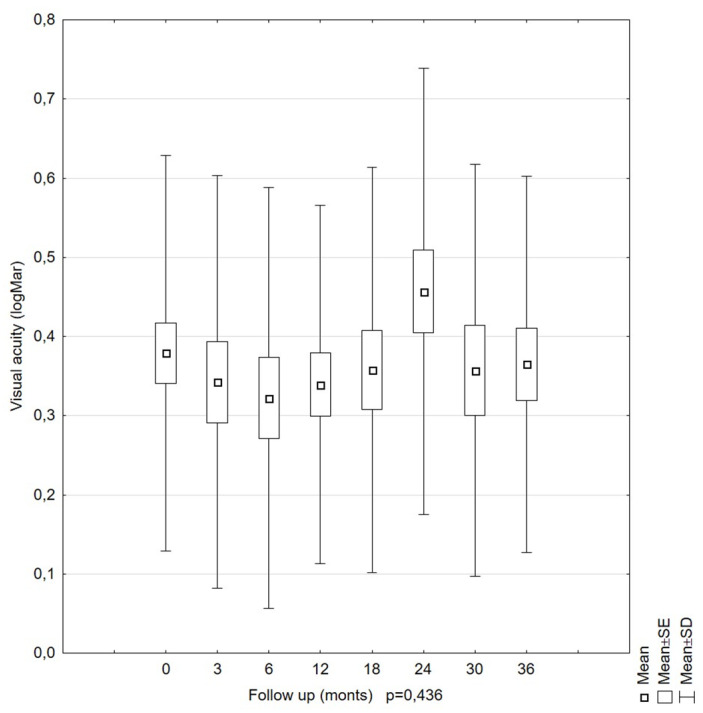
Mean best corrected visual acuity (BCVA) values in LMH patients during the 36-month follow-up period. The BCVA remained stable over the entire follow-up period.

The OD parameter was also evaluated. As for the previous LHM parameters, no significant changes over time were observed. The mean OD measurements were 1132.26 µm (SD = 477.29) at the first examination and 1119.60 µm (SD = 451.22) at the last examination. As for the previous LHM parameters, no significant changes over time were observed (*p* = 0.985). The calculated mean values were similar. The highest mean value was recorded at 3 months, and the lowest, at 0 months (the first examination). Errors for mean values were the highest at 3 months, while the lowest confidence intervals for the mean values in subsequent measurements were similar (Figure 7).

**Figure 8:**
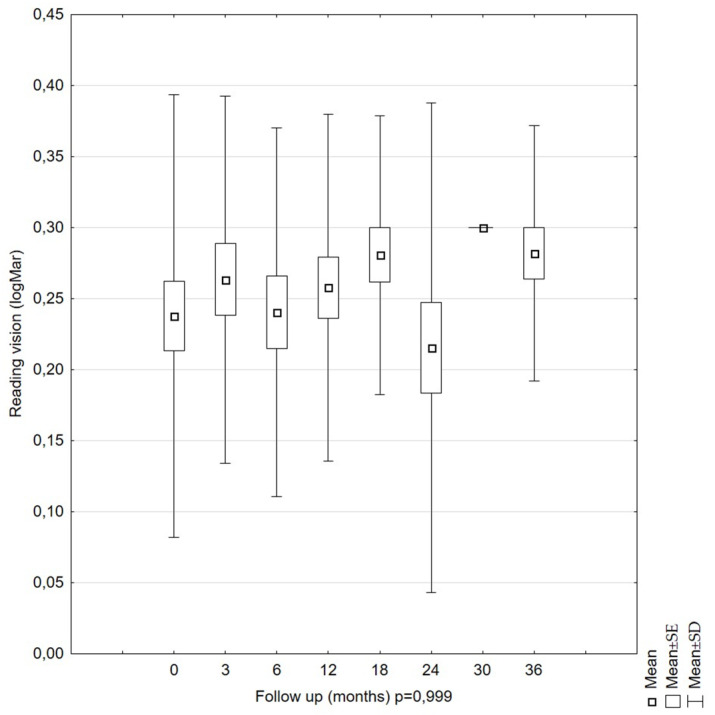
Mean reading vision (RV) values in LMH patients during the 36-month follow-up period. The RV remained stable over the whole follow-up period.

**Figure 9:**
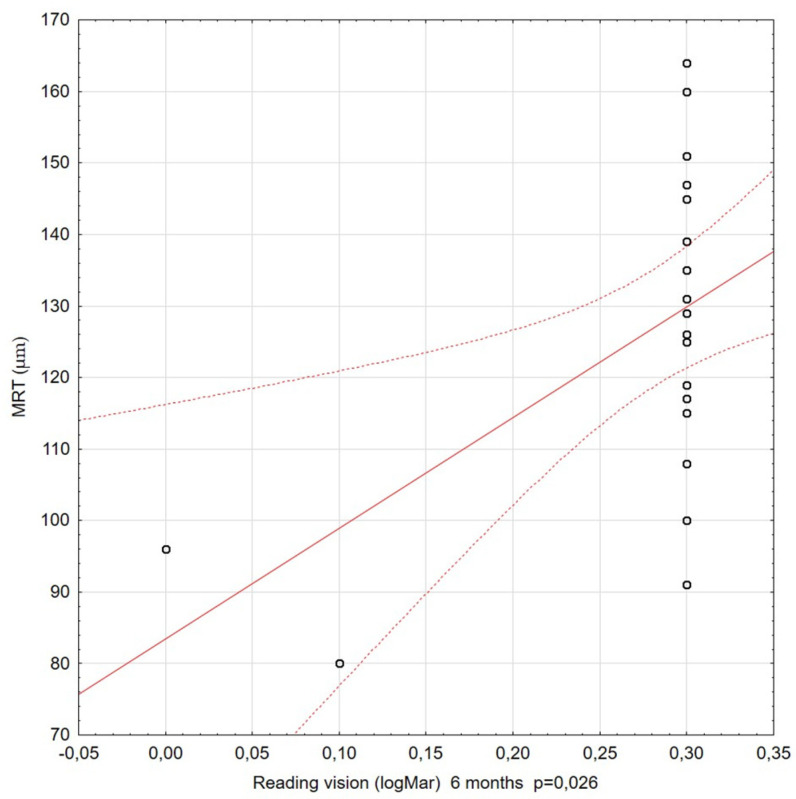
Correlation between reading vision (RV) (at 6 months) and minimal retinal thickness (MRT). A statistically significant correlation was observed: better RV correlated with higher MRT values.

The analyzed parameters, such as visual acuity (*p* = 0.435) and reading vision (*p* = 0.0999), were stable, and there was no indication of significant differences according to the time of the examination within the 36-month follow-up period (Figure 8 and Figure 9). The mean visual acuity at the beginning of the follow-up was 0.38 (logMar) (SD = 0.25), and that at the end was 0.36 (logMar) (SD = 0.24). The mean reading vision at the beginning of the study was 0.24 (logMar) (SD = 0.16), and that at the end was 0.28 (logMar) (SD = 0.09).

**Figure 10:**
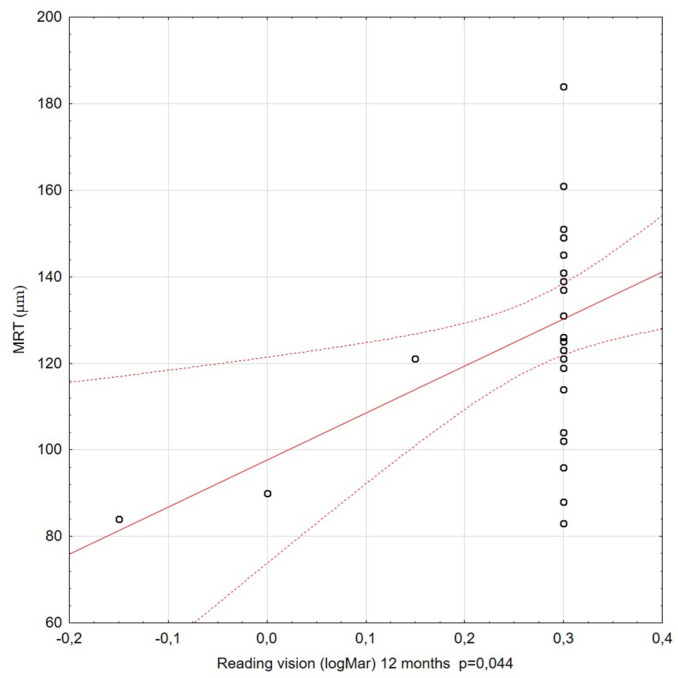
Correlation between reading vision (RV) (at 12 months) and minimal retinal thickness (MRT). A statistically significant correlation was observed: better RV correlated with higher MRT values.

**Figure 11:**
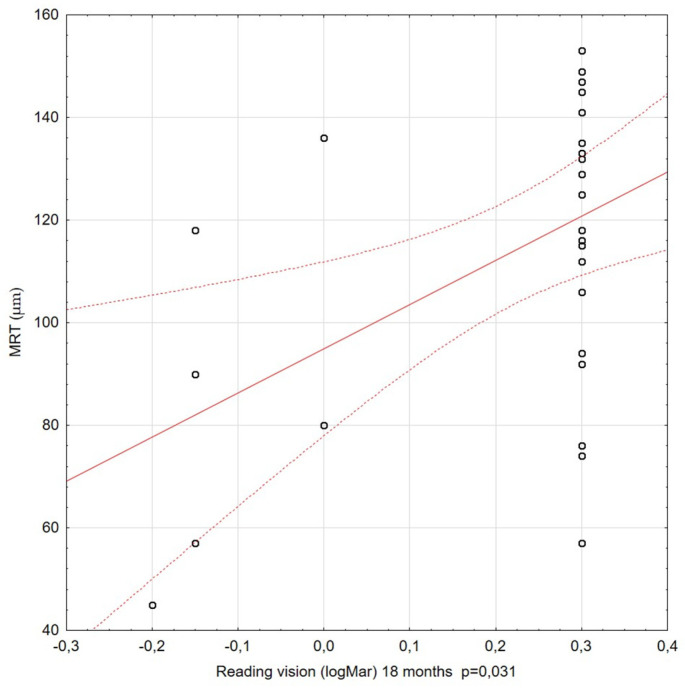
Correlation between reading vision (RV) (at 18 months) and minimal retinal thickness (MRT). A statistically significant correlation was observed: better RV correlated with higher MRT values.

The correlations among visual acuity, reading vision, and MRT were also assessed at subsequent stages of the study. For most of the Spearman’s rank correlation coefficients calculated during the 36-month observation period, there was no significant correlation between the BCVA and MRT. Significant relationships between MRT and RV were only relevant for observations at 6, 12, and 18 months of the study follow-up period. The coefficient values were moderate and positive. Better reading vision coexisted with higher residual retinal thickness (Figure 10 and Figure 11).

## 4. Discussion

Our study was conducted on a group of 40 patients aged 50 to 78 (mean age, 67.87; SD = 10.14) over a 36-month follow-up period. To the best of our knowledge, this is one of the largest groups of LMH patients observed over a long period. Most of the patients were women (87.50%). The age structure of our patients, examinations, time intervals, criteria of morphological evaluation, and equipment used were similar to those in other studies [18,23,24,25,26,27,28].

Morphological parameters such as the MRT, Max RT temporal, Max RT nasal, ID, and OD were stable over the 36-month follow-up period for the LMH patients. Similar results for the morphological parameters (OD, MRT, Max RT temporal, and Max RT nasal) in LMH patients were shown by Garcia-Fernandez [29].

One of the most important parameters analyzed was the minimal retinal thickness (MRT). The change in this value was not statistically significant during the long-term follow-up, which was also consistent with the available findings in the literature [25,29]. In our group, the mean values of the MRT measurement were 117.68–122.35 µm, and were stable over time, as compared with those of Gaudric et al., who, analyzing a group of 29 eyes with LMH, obtained much lower mean MRT results (70 ± 18 µm) [13]. By contrast, Bottoni observed significantly higher MRT values in patients with LMH (180 ± 29 µm) [25]. Nevertheless, both studies confirmed a lack of significant variability during long-term follow-up.

According to Parravano, disorders in the outer LMH layers are caused by vitreomacular adhesion (VMA) and posterior vitreous detachment (PVD) [27]. The thinning of the retina within the LMH area (MRT) is the result of the mechanism by which this pathology manifests, where the separation of the cyst roof within the fovea can occur during the shrinking of the vitreous humor (PVD) without the opening of the outer layers of the retina. This has been confirmed by Gaudric and others, who reported the presence of a pseudo-lid in a biomicroscopic examination and OCT examination. This confirms that the lamellar macular hole may be disrupted in the formation of a full-thickness macular hole (FTMH) [30,31,32]. The MRT can also be affected by the epiretinal membrane (ERM). These conclusions were suggested by Bottoni during an evaluation of ERM’s influence on LMH progression [25]. In our study, the observed mean values of the Max RT temporal and Max RT nasal parameters were stable during the 36-month follow-up period. The mean values of the MaxRT temporal measurement were 327.58–333.39 µm, and the mean values of the Max RT nasal measurement were 347.41–348.47 µm. These results were similar to those observed in other studies. In a study carried out by Parravano et al., these values were, respectively, a Max RT temporal of 341.9 ± 49.9 µm and Max RT nasal of 337.5 ± 55.9 µm [27]. Lower values were obtained by Gaudric et al. In a study group of 29 eyes with LMH, the mean Max RT temporal was 279 ± 40 µm, and the mean Max RT nasal was 287 ± 37 µm. Similar to our study, both studies showed these parameters to be stable over time [13]. Certain discrepancies between our measurements and other authors’ measurements were observed, which may be due to the type of OCT device used and the test methodology (manual measurement). In addition, several mechanisms may be responsible for the thickening of the paracentral retina around the hole. Vitreomacular adhesion (VMA), associated with PVD, may lead to the spreading of the retinal layers with subsequent thinning in the center. The available literature has also documented cases of macular holes without the participation of vitreoretinal traction in eyes with total, long-lasting PVD. Therefore, it can be assumed that these holes were formed by a mechanism other than a traction mechanism. Such a state could be explained by microdamage in the continuity of the inner limiting membrane (ILM), which, by causing swelling of the retina, led to the spreading of the central retinal layers and tangential traction caused by the glial membrane adjacent to the retina [33]. Moreover, simultaneous damage to the Müller cells enlarges the area of retinal edema in the fovea, which creates a force acting tangentially to the retinal surface. This centrifugal force leads to the spreading of the layers around the fovea. A shrinking glial membrane stratifies the plexiform layers, causing the formation of intraretinal cysts, which leave the thinned retina in the fovea after bursting.

Another parameter analyzed in this study was the LMH internal diameter (ID). A slight increase in the ID was found between the measurements at 0 and 36 months. The mean IDs were 450.42 µm (SD = 208.60) at the first examination and 498.76 µm (SD = 184.52) at the last examination. A comparison of the values obtained in our study with the LMH results obtained by Gaudric indicated that the ID values in our patients were about 100 µm lower (in Gaudric’s study, ID = 569 ± 159 µm) [13]. Similar to the case of the MRT and Max RT temporal, the source of the discrepancy lies in the methodology for the applied measurements. Michalewska et al. examined patients with LMH and obtained ID values that depended on the resolution of the OCT device, and ranged from 537 µm (Copernicus OCT) to 684 µm (Spectralis OCT). These values did not change in a statistically significant manner [5].

Our results are consistent with those of Garcia-Fernandez [29]. However, they are not consistent with those of Bottoni. Our estimated parameters were slightly higher. The OD values in Bottoni’s studies ranged from 230 to 1010 µm. Additionally, Bottoni obtained statistically significant OD changes over time in a group of seven eye pairs [25]. The differentiation in the OD results found by Bottoni may have resulted from different scanning positions during the first and subsequent follow-up examinations. Theodossiadis et al. observed OD values slightly higher than those observed by Bottoni [33]. The OD values ranged from 624.54 ± 191.15 µm at the beginning of the observation to 710.22 ± 210 µm at the last examination. The variability of the OD parameter over time proved to be statistically significant (*p* = 0.001), similar to Bottoni’s findings. The percentage of people with an increasing OD was smaller than that observed by Bottoni (13.7% vs. 21%). Theodossiadis et al. found a correlation between an increase in OD and the presence of ERM. LMH patients with ERM showed an increase in OD of more than 10% compared to the LMH patients without ERM. Clamp and Witkin also showed no significant differences in the LMH OD value between the ERM and no-ERM patients [34,35]. In our study, the conducted analysis demonstrated that LMH was accompanied by stable RV and BCVA over the 36-month follow-up period, and the results confirmed previous observations made by other authors [23,24,25,26,29].

Bottoni et al. observed LMH in 34 patients aged 54 to 87 years (mean age, 73) over a period of 18 months and demonstrated that the corrected visual acuity (V) was 63 ± 6 letters and did not change significantly over time (*p* = 0.256) [25]. A similar correlation over time was observed by Garcia-Fernandez [29].

BCVA is closely related to the morphological condition of LMH. The morphological conditions of LMH and BCVA were both stable during the long-term follow-up. In the literature on LMH, only BCVA has been assessed, and therefore, no data are available that confirm similar stability with respect to RV. Our studies indicated such a tendency.

In our study, we did not observe any significant relationship between MRT and BCVA, which was partly confirmed by Parravano’s study [27]. Theodossiadis indicated a positive and significant relationship between the parameters (*r* = 0.47, *p* = 0.002) [33]. Similar observations were made by Bottoni et al.

The main finding of our study is the relationship between MRT and RV. To the best of our knowledge, the relationship between RV and MRT has not been studied, quite surprisingly, since most macular diseases affect RV with the presence of metamorphopsia. Although the correlation between RV and MRT was weak, it was statistically significant (*r* = 0.32, *p* = 0.000). Therefore, it should be considered, especially if a decision about a surgical intervention is to be made. In our experience, LMHs with high MRT values and a lack of metamorphopsia were stable, with a low progression rate; therefore, vitrectomy should be considered with caution and only in cases with significant macular morphology and functional decline.

The major limitations of the study include the small group examined, the lack of autofluorescence images showing the alteration in RPE, and, finally, the lack of an angio-OCT examination. We believe that impaired blood flow in degenerative LMHs may be a major risk factor for surgical failure, but clinical trials are needed to confirm this theory. The approaches for treating LMHs are very likely to change in the future due to a better understanding of their background and various morphological forms.

## Data Availability

All the data will be made available upon reasonable request from the corresponding authors.
